# Efficacy of sacubitril-valsartan combined with rosuvastatin calcium in the treatment of unstable angina pectoris and its effects on blood lipids and hemorheology

**DOI:** 10.12669/pjms.41.5.10658

**Published:** 2025-05

**Authors:** Yang Peng, Yuxiang Wen, Han Wei

**Affiliations:** 1 Yang Peng Department of Cardiology, The First Affiliated Hospital of Yangtze University, Jingzhou 434000, Hubei, China; 2 Yuxiang Wen Department of Cardiology, The First Affiliated Hospital of Yangtze University, Jingzhou 434000, Hubei, China; 3 Han Wei Department of Cardiology, The First Affiliated Hospital of Yangtze University, Jingzhou 434000, Hubei, China

**Keywords:** Unstable angina pectoris, Sacubitril-valsartan, Rosuvastatin calcium, Efficacy

## Abstract

**Objective:**

To investigate the clinical efficacy of sacubitril-valsartan (SV) combined with rosuvastatin calcium (RC) in the treatment of patients with unstable angina pectoris (UAP).

**Methods:**

This was retrospective study. Eighty patients with UAP admitted to The First Affiliated Hospital of Yangtze University from December 2022 to December 2023 were included and divided into observation group (n=40) and control group (n=40) according to treatment regimens. Patients in the control group received RC, while the observation group received SV combined with RC. Clinical efficacy, adverse reactions, and blood lipid, hemorheology, and cardiac function before and six months after treatment were compared between the two groups.

**Results:**

The total efficacy of the observation group was higher than the control group (P<0.05). After six months of treatment, the levels of TG, TC, and LDL-C decreased in both groups, with the observation group showing more significant decrease, while the HDL-C level increased, with the observation group showing more significant increase (P<0.05); The hemorheological indicators of both groups decreased compared to before treatment, and the degree of decrease was more significant in the observation group (P<0.05); Both groups showed significant improvement in cardiac function levels compared to before treatment, with the observation group showing more pronounced improvement (P<0.05). The incidence of adverse reactions in both were not statistically significant different (P=0.712).

**Conclusion:**

The use of SV combined with RC in treatment of UPA has significant clinical effects, improving patients’ blood lipids, hemorheology, and cardiac function indicators without increasing adverse reactions.

## INTRODUCTION

Cardiovascular disease (CVD) is one of the major diseases threatening human life and health and also the leading cause of death. The CVD burden raised to about 523.2 million new cases and 18.6 million deaths worldwide in 2019.^1^ The incidence of CVD has increased by 17.1% in the past decade, and currently, there are about 330 million patients with CVD in China, of which about 11.392 million patients suffer from coronary heart disease (CHD).^2^ In 2020, CVD is responsible for 48.00% and 45.86% of deaths in rural and urban areas, respectively, in China.

The incidence of CVD has posed a heavy economic burden to patients and society and become a major public health issue of social concern.^3^ CHD is classified into chronic coronary artery disease (CAD) and acute coronary syndrome (ACS), and unstable angina pectoris (UAP) is a subtype of ACS. The development of UAP is unpredictable, and timely and effective treatment may not be provided, leading to serious cardiovascular events such as acute myocardial infarction (AMI), heart failure, and even sudden death in severe cases.^4^

The pathogenesis of UAP is complex, which is mainly related to factors such as abnormal blood lipid profile and hemorheology, and secondary thrombosis. No consistent treatment regimens are currently available for UAP. The treatment goal includes early clinical intervention to stabilize plaque, improve cardiac function and prevent the progression of the disease. Medical treatment is often used to improve circulatory disorders and coronary ischemia and protect cardiac function. Rosuvastatin calcium (RC) is a commonly used drug for the treatment of UAP, which controls the progression of UAP via regulating blood lipids and stabilizing plaques.^5^

Sacubitril-valsartan (SV), the only angiotensin receptor-neprilysin inhibitor approved for marketing, works through maintaining the level of natriuretic peptide and blocking the renin-angiotensin-aldosterone system (RAAS) and the sympathetic nervous system (SNS).^6^ Clinical application of SV and RC has been widely reported, and the combination of these drugs in the treatment of UAP, however, has not been documented. On this basis, to investigate the clinical efficacy of SV combined with RC in the treatment of patients with UAP was retrospectively analyzed in the present study.

## METHODS

This was retrospective study. Eighty patients with UAP admitted to The First Affiliated Hospital of Yangtze University from December 2022 to December 2023 were recruited as subjects and divided into the observation group (received sacubitril-valsartan combined with rosuvastatin calcium) and the control group (received with rosuvastatin calcium) according to the treatment regimens they received, with 40 patients in each group. There were 24 males and 16 females in the observation group, with an age of 25-62 (52.48±9.74) years old and a disease course of 1-6 (3.55±1.57) years, and 26 males and 14 females in the control group, with an age of 27-65 (51.65±10.07) years old and a disease course of 1-8 (3.85±1.83) years. The two groups were comparable with no statistically significant differences in the general data (P>0.05).

### Ethical Approval:

This study was approved by the Institutional Ethics Committee of the First Affiliated Hospital of Yangtze University (No: KY2024-058-01; Date: August 23, 2024), and all patients and their families were informed and consented to participate in this study.

### Inclusion criteria:

- Who met the diagnostic criteria of UAP with low or medium risks?
- With definite evidence from ECG and cardiac enzyme tests, without AMI.
- With an age of 18-80 years old.

### Exclusion criteria:

- Who were allergic to drugs used in the study?
- With severe hepatic and renal dysfunction, infectious diseases, and mental disorders.
- With AMI.
- With severe abnormalities in coagulation function.
- Who were unable to cooperate with the treatment, with incomplete clinical data?

On the first day after admission, all patients received health education and appropriate exercise training, and conventional anticoagulation, antihypertensive and antiplatelet aggregation treatments. Patients in the control group was also treated with oral RC [AstraZeneca Pharmaceutical (China) Co., Ltd., approval number: Guo Yao Zhun Zi J20170008, strength: 10 mg], 10mg, once daily after dinner, and those in the observation group were treated with oral SV (Novartis Pharma Schweiz AG, registration number: H20171198, 100 mg), 100 mg, twice daily, in addition to RC. The dose of SV increased to 200 mg, twice daily after two weeks of treatment. Patients in two groups were treated for three months. The follow-up time for patients in both groups was six months.

Clinical efficacy was evaluated as markedly effective (the resting electrocardiogram returned to normal or the exercise test turned negative after treatment, and clinical symptoms such as pain resolved), effective (the frequency of attacks significantly decreased and clinical symptoms improved after treatment), and ineffective (patients failed to meet the above diagnostic criteria). Total effective rate was calculated as the sum of rates of markedly effective and effective.

### Outcome Measures:

- Blood lipid parameters: Fasting venous blood was collected from patients before and six months after treatment, and triglyceride (TG), total cholesterol (TC), high-density lipoprotein cholesterol (HDL-C) and low-density lipoprotein cholesterol (LDL-C) were determined using an automatic biochemical analyzer;
- Fasting venous blood was collected before and six months after treatment, and whole blood high/low shear viscosity, plasma viscosity and fibrinogen were detected using an automatic hemorheometer;
- Cardiac function, including left ventricular end-diastolic dimension (LVEDD), left ventricular end-systolic dimension (LVESD) and left ventricular ejection fraction (LVEF), were measured for the two groups of patients before and six months after treatment using LOGIQ-500 color Doppler ultrasound (GE, USA); (
- The incidence of adverse reactions, including gastrointestinal(GI) bleeding, nausea and vomiting, dizziness and headache, muscle soreness, and fatigue, in the two groups was recorded to evaluate the safety of medication.

### Statistical Analysis:

Data were analyzed using SPSS22.0 software. Measurement data were presented as mean ± standard deviation (*χ̅*±*S*), and t test was used for comparison. Enumeration data were presented as n (%), and χ² test was used for comparison. Differences with a p value of <0.05 were considered statistically significant.

## RESULTS

The total efficacy of the observation group was 95.00%, which was higher than that of the control group (80.00%), and the difference was statistically significant (χ²=4.114, P<0.05) (Table-I). No significant differences in the levels of TC, TG, LDL-C and HDL-C were observed between the two groups before treatment (P>0.05); and six months after treatment, the levels of TG, TC and LDL-C in the two groups decreased, and the levels of these parameters were significantly decreased in the observation group compared with those in the control group. The level of HDL-C increased in the two groups, and the level was significantly increased in the observation group compared with that in the control group. The differences between the two groups were statistically significant (P<0.05) (Table-II).

**Table-I:** Comparison of clinical efficacy between the two groups[n(%)].

Groups	n	Markedly effective	Effective	Ineffective	Total effective rate
The observation group	40	34(85.00)	4(10.00)	2(5.00)	39(95.00)
The control group	40	29(72.50)	3(7.50)	8(20.00)	34(80.00)
*χ* ^2^					4.114
*P*					0.043

**Table-II:** Comparison of blood lipid parameters between the two groups before and after treatment(*χ̅*±*S*).

Groups	n	TG(mmol/L)		TC(mmol/L)		LDL-C(mmol/L)		HDL-C(mmol/L)	
		Before treatment	6 months after treatment	Before treatment	6 months after treatment	Before treatment	6 months after treatment	Before treatment	6 months after treatment
The observation group	40	5.25±0.68	2.66±0.51	8.10±0.93	5.50±0.84	4.68±0.56	2.51±0.61	1.22±0.37	2.52±0.48
The control group	40	5.29±0.73	3.54±0.65	8.15±0.87	6.43±0.82	4.85±0.70	3.08±0.66	1.28±0.37	1.90±0.44
*t*		0.302	6.787	0.261	5.000	1.211	4.009	0.628	6.017
*P*		0.764	0.000	0.795	0.000	0.229	0.000	0.532	0.000

There were no significant differences in whole blood high/low shear viscosity, plasma viscosity and fibrinogen between the two groups before treatment (P>0.05); and six months after treatment, the whole blood high/low shear viscosity, plasma viscosity and fibrinogen decreased in the two groups, and the improvement in the observation group was better than that in the control group. The differences between the groups were statistically significant (P<0.05) (Table-III).

**Table-III:** Comparison of hemorheological parameters between the two groups(*χ̅±s*).

Groups	n	Whole blood high shear viscosity(mpa/s)			Whole blood low shear viscosity(mpa/s)			Plasma viscosity (mpa/s)		Fibrinogen(g/L)	
		Before treatment	6 months after treatment	Before treatment		6 months after treatment	Before treatment		6 months after treatment	Before treatment	6 months after treatment
The observation group	40	9.38±0.96	4.04±0.61	14.53±2.49		8.13±2.18	2.35±0.27		1.33±0.76	5.27±0.76	2.05±0.31
The control group	40	9.47±0.33	4.94±0.63	14.51±2.24		9.92±2.64	2.32±0.31		1.74±0.42	5.19±0.35	3.58±0.59
*t*		0.566	7.59--959	0.036		3.307	0.457		2.978	0.634	14.454
*P*		0.573	0.000	0.971		0.001	0.649		0.004	0.528	0.000

No significant differences in LVEDD, LVESD and LVEF were observed between the two groups before treatment (P>0.05); and after treatment, the levels of LVEDD, LVESD and LVEF in the two groups were improved compared with those before treatment (P<0.05), and the improvement of these parameters were better in the observation group than that in the control group, with statistically significant differences (P<0.05) (Table-IV).

**Table-IV:** Comparison of cardiac function between the two groups before and after treatment(*χ̅±s*).

Groups	LVEF(%)		LVEDD(mm)			LVESD(mm)	
	Before treatment	After treatment	Before treatment	After treatment	Before treatment		After treatment
The observation group	41.53±4.28	58.35±4.06	60.30±3.61	46.93±3.26	56.30±3.62		42.83±3.12
The control group	41.38±4.09	53.10±3.75	59.80±3.82	54.23±3.61	55.30±3.73		48.73±3.75
*T*	0.160	6.004	0.602	9.487	1.217		7.583
*P*	0.873	0.000	0.549	0.000	0.227		0.000

Adverse reactions were reported in both groups during treatment. The incidence of adverse reactions in the observation group was 7.50%, which was lower than that in the control group (12.50%), and the difference was not statistically significant (χ²=0.556, P=0.712) (Table-V).

**Table-V:** Comparison of adverse reactions between the two groups [n(%)].

Groups	n	GI bleeding	Nausea and vomiting	Dizziness and headache	Muscle soreness	Fatigue	Total incidence
The observation group	40	1(2.50)	1(2.50)	0(0.00)	0(0.00)	1(2.50)	3(7.50)
The control group	40	0(0.00)	1(2.50)	2(5.00)	1(2.50)	1(2.50)	5(12.50)
χ^2^							0.556
*P*							0.712

## DISCUSSION

UAP is a subtype of ACS characterized by sudden onset and easy recurrence.^7^ The goal of treatment is to improve stability of coronary plaque and hemorheology, reduce blood lipids, and prevent platelet aggregation. The results of this study showed that the total clinical efficacy of the observation group was 95.00%, which was higher than the control group’s 80.00% (χ²=4.114, P<0.05). The main reason for the analysis is that the pathogenesis of UAP is complex, and single drug therapy cannot achieve the ideal effect of controlling the disease. Previous studies have shown that the highest effective rate of single drug therapy is 70%, while combination therapy can achieve better clinical results in the treatment of UAP.

Our research results also confirm this point.^10^ Patients with UAP often have dyslipidemia, and lipid-lowering therapy is one of the main measures for the prevention and treatment of UAP. The results of this study showed that after six months of treatment, the levels of TG, TC, and LDL-C in both groups decreased and the observation group was lower than the control group, while the levels of HDL-C increased and the observation group was higher than the control group (P<0.05), indicating a synergistic effect of SV combined with RC. In the past, a large number of studies have confirmed that the levels of TC and LDL-C are significantly and positively correlated with the incidence of UAP,^11^,^12^ while the increase of HDL-C has the benefit of anti-atherosclerosis, and its decrease can be used as a factor to predict cardiovascular events. The better lipid-lowering mechanism of SV combined with RC is mainly due to the fact that RC belongs to the statin class, with a long half-life, high utilization, and fast onset, and can inhibit cholesterol synthesis; The valsartan in SV is an Ang II receptor type 1 (AT1) receptor blocker, which improves lipid metabolism by promoting the normal generation and differentiation of adipocytes.^13^,^14^

Improving the hemorheological state is an effective measure for preventing and treating UAP. The results of this study showed that after treatment, the whole blood high/low shear viscosity, plasma viscosity, and fibrinogen of both groups of patients significantly decreased, and the improvement effect of the observation group was better than that of the control group (P<0.05). This is similar to previous research results, indicating that the combination of SV and RC can better improve the hemorheological indicators of UAP patients. UAP patients often exhibit significant abnormalities in hemorheological indicators, mainly manifested as increased blood viscosity and slow blood flow,^15^ RC can mobilize endothelial cells, coagulation factors, anticoagulant proteins, etc., improve the pre thrombotic state, stabilize plaques, and alter hemorheological indicators;^16^ SV regulates water sodium balance, dilates blood vessels, and regulates changes in blood rheology by acting on the renin angiotensin aldosterone system.^17^

The results of this study showed that after treatment, the levels of LVEDD, LVEBD, and LVEF in both groups of patients improved compared to before treatment, and the improvement in the observation group was more significant (P<0.05). Previous studies have also shown that the combination of SV guidelines can improve cardiac function.^18^ The analysis suggests that RC can improve endothelial function and enhance myocardial cell tolerance,^19^ while SV can reduce myocardial cell apoptosis, prevent ventricular fibrosis, and alleviate myocardial vascular remodeling, the combined application of the two can play a complementary and synergistic role.^20^,^21^ This study analyzed its safety, and the results showed that there was no statistically significant difference in the incidence of adverse reactions between the two groups of patients (χ²=0.556, P=0.712), indicating that the use of SV combined with RC for the treatment of unstable angina is safer.

### Limitations

This was a single-center study with small sample size and the long-term efficacy and safety were not observed, and future studies are needed.

## CONCLUSIONS

Sacubitril-valsartan combined with RC in the treatment of patients with UAP can significantly improve blood lipid and hemorheological parameters and increase the clinical efficacy and cardiac function, with a favorable safety profile.

### Authors’ Contributions:

**HW:** Carried out the studies, participated in collecting data, and drafted the manuscript, and are responsible and accountable for the accuracy or integrity of the work. **YP:** Collected and analyzed clinical data. Critical Review. **YW:** Literature search, Performed the statistical analysis and participated in its design. All authors read and approved the final manuscript.
